# Editorial: Cytokines in inflammatory, infectious and noninfectious diseases

**DOI:** 10.3389/fimmu.2026.1927415

**Published:** 2026-08-11

**Authors:** Geovane Dias-Lopes, Thiago DeSouza-Vieira, Danielle Aparecida Sousa Rodrigues, Rafael Cardoso Maciel Costa Silva

**Affiliations:** 1 Faculdade de Ciências Médicas, Universidade do Estado do Rio de Janeiro, Cabo Frio, Rio de Janeiro, Brazil; 2 Laboratório de Bioquímica e Fisiologia de Insetos, Instituto Oswaldo Cruz, Fundação Oswaldo Cruz, Rio de Janeiro, Brazil; 3 Laboratório de Biologia de Linfócitos, Instituto de Biofísica Carlos Chagas Filho, Universidade Federal do Rio de Janeiro, Rio de Janeiro, Brazil

**Keywords:** cytokines, infectious diseases, inflammation, tissue damage, tissue repair

Cytokines are soluble extracellular proteins or glycoproteins that orchestrate pivotal intercellular signaling networks, integrating immune and non-immune cell responses to infection, tissue injury, metabolic disturbances and environmental stress across multiple organs and tissues. They function as a dynamic communication network rather than acting in isolation, coordinating the initiation, amplification, and resolution of immune responses while maintaining tissue homeostasis. Beyond their classical role in immunity, their influence extends to essential processes such as hematopoiesis, angiogenesis, tissue remodeling, neurotransmission circuits and metabolism. This remarkable versatility is defined by their pleiotropic and context-dependent nature, such that the ultimate biological effect is shaped by a complex interplay of redundancy, synergism, and antagonism among cytokine families. By acting in this manner, these chemical mediators determine the delicate balance between mounting effective responses against pathogens, tumor cells, and other danger signals, while simultaneously limiting excessive inflammation and promoting tissue repair. In this context, pro-inflammatory cytokines sustain inflammation and may contribute to tissue damage; whereas anti-inflammatory cytokines suppress immune responses and, under certain circumstances, facilitate pathogen persistence. Increasing evidence highlights the intimate link between cytokines and cellular metabolism, fostering the rapidly expanding field of immunometabolism. In this paradigm, metabolic pathways regulate cytokine production, while cytokines, in turn, orchestrate the reprogramming of cellular metabolism. Consequently, dysregulated cytokine production—whether excessive, persistent, or insufficient—can drive immunopathology rather than protection and has been implicated in the pathogenesis of a broad spectrum of inflammatory, infectious, and noninfectious diseases. Of note, inflammatory diseases occur when the primary cause of the homeostasis disturbance is abnormal inflammation, which includes autoimmunity, dysbiosis and genetic disorders that affect genes involved in the immune response regulation. Infectious diseases are caused by pathogens, such as SARS-CoV-2 and the hepatitis B virus, which are inherently associated to an inflammatory reaction to control infection. While non-infectious diseases are primarily driven by other mechanisms that do not involve pathogens or an intrinsic immune dysregulation, for example, diabetes (caused by deficient insulin production or signaling), inflammatory damage and an impaired immune response are often part of the pathogenesis. The remarkable clinical success of cytokine-targeting biologics, immune checkpoint therapies, and cytokine-based biomarkers has transformed cytokine biology from a fundamental area of immunology into a cornerstone of precision medicine. This Research Topic, focused on cytokines in inflammatory, infectious, and noninfectious diseases, brings together a diverse collection of studies highlighting their dual role as essential mediators of host defense and drivers of disease, as well as unconventional modulators of cytokine production and their potential utility as clinical biomarkers, prognostic tools, and therapeutic targets.

Portraying novel biological roles for cytokines and the complex dynamics of IFNs pathways, a review synthesizes the double-edged role of type I interferons during bacterial infections, in which Lyadova highlights how these cytokines can either promote host defense or drive pathology by reprogramming the myeloid cell compartment, specifically by altering macrophage metabolism, inducing cell death, and modulating emergency myelopoiesis. Meanwhile, investigating systemic autoimmunity in patients from Western India, Khatri et al. demonstrate that activation of all three interferons (IFNs) pathways in Systemic Lupus Erythematosus (SLE) closely correlates with distinct clinical manifestations, supporting the clinical utility of IFN-based patient stratification. On the other hand, clinical intervention with IFNs may also trigger severe immune dysregulation. Wang et al. describe a rare case report of hemophagocytic lymphohistiocytosis developing in a patient with chronic hepatitis B following treatment with synthetic cytokine IFN-α-2b complexed with polyethylene glycol (PegIFN-α-2b), illustrating how IFN therapy may unexpectedly precipitate life-threatening hyperinflammation and subsequent opportunistic infections. These findings expand the important roles of interferons in immunity, and clinical setting of SLE and therapies based on this cytokine.

Beyond IFNs, several studies extend the application of distinct cytokines to the inflammatory milieu of neurological disorders, inflammatory diseases, immune-metabolic interactions, and precision diagnoses. Thaçi et al. review how site-specific glycosylation patterns modulate IgE activity beyond traditional allergies, uncovering novel therapeutic targets within IgE glycosylation, which influences immune cells activation and secretion of cytokines, like IL-13 and IL-4. The authors also discuss the role of cytokines produced by follicular T cells in promoting IgE class-switch recombination in activated B cells. Li et al. conduct a retrospective multicenter study demonstrating that baseline neutrophil counts and the platelet–lymphocyte ratio serve as simple and cost-effective predictors of clinical response to ustekinumab (a monoclonal antibody targeting the p40 subunit of IL-12 and IL-23) in patients with Crohn’s disease, offering a practical tool to optimize inflammatory bowel disease management with accessible clinical biomarkers for personalized medicine. Duray et al. combine multiplex cytokine profiling with machine learning and linear regression to predict both disease etiology and hospitalization time in pediatric lower respiratory infections, highlighting the growing potential of immune signatures for clinical decision-making. Addressing neuroinflammation, Yang et al. uncover aberrant metabolic signatures of long-chain fatty acids (LCFA) during relapsing-remitting multiple sclerosis relapses, establishing potential LCFA biomarkers while mapping their interaction with peripheral cytokines and chemokines, including IL-7, IL-17A, TNF-α, CCL2, and CXCL8. Expanding the paradigm of neurovascular inflammation, Bai et al. reveal diffuse inflammatory pathway activation in patients with cerebral autosomal dominant arteriopathy with subcortical infarcts and leukoencephalopathy, demonstrating that elevated peripheral cytokines, particularly TNF-β (Lymphotoxin-α), correlate closely with worse cognitive performance, apathy, and a higher burden of deep and lobar cerebral microbleeds. Collectively, these studies illustrate the growing value of integrated cytokine and immunometabolic signatures as biomarkers for disease stratification, prognosis, and therapeutic guidance across diverse clinical settings.

Moving from biomarker discovery toward therapeutic intervention, several contributions illustrate how the indirect modulation of inflammatory pathways can improve clinical outcomes. In this sense, Zhang et al. demonstrate that a change in the material, i.e., plastic cannulas, can outperform metal needles in arteriovenous fistulas by suppressing Complement Factor B, thereby reducing localized inflammation and preventing vascular access failure. A similar emphasis on reprogramming localized immune responses to drive tissue healing is found in regenerative orthopedics. Li et al. discuss recent advances demonstrating how mesenchymal stem cells (MSCs) act as promising therapeutic agents in rotator cuff injuries by establishing bidirectional *crosstalk* with host immune cells that reshapes the inflammatory microenvironment toward regenerative healing. Beyond differentiation into tenocytes, chondrocytes, and osteoblasts, MSCs exert potent paracrine effects through secretion of TGF-β, VEGF, IGF-1, and IL-6, and numerous bioactive mediators that promote tendon-to-bone repair. Furthermore, authors also highlight AI-driven stratification of cytokine profiles as the future of personalized regenerative interventions. In summary, these studies demonstrate that distinct approaches, material and cellular-based, can improve the management of non-infectious pathologies through the optimization of the inflammatory response.

Expanding on surgical interventions and reproductive health, Jeong et al. investigate the local microenvironment in cervical insufficiency, detailing the dynamic shifts in soluble immune-checkpoint regulators and inflammatory cytokines/chemokines before and after cervical cerclage. Their multivariate model demonstrates that combining elevated pre-cerclage IL-17A with decreased post-cerclage PD-L1 yields excellent prognostic performance in predicting subsequent preterm birth. These studies underscore both the risk and the impact of cytokines in the treatment of infectious diseases and in reproductive health.

The Research Topic further expands into mechanistic studies that deepen our understanding of how cytokine networks orchestrate inflammation through complex interactions with cellular metabolism and tissue homeostasis. Vella et al. offer a critical synthesis of the immunopathogenesis of sepsis, focusing on the precise molecular and cellular mechanisms that govern cytokine regulation during systemic collapse, discussing the critical role of distinct cytokines and chemokines like TNF-α, IL-6, GM-CSF, IFN-γ, TGF-β, CCL1, CCL2, and CXCL8. Furthermore, providing specific mechanistic insights into bacterial outcomes during early development, Annamanedi et al. demonstrate that the absence of IL-27 signaling improves survival in neonatal *Escherichia coli* sepsis by enhancing protective immune-cell recruitment and bacterial clearance. Together, these contributions reinforce the context-dependent nature of cytokine signaling during infection, where identical cytokine pathways may either protect the host or contribute to disease progression during the early or adult stage. The understanding of the mechanisms involved in the specific role of cytokines in infectious diseases can lead to the development of compelling therapeutic interventions.

The importance of cytokine networks is equally evident in viral diseases. Wolszczak-Biedrzycka et al. review how SARS-CoV-2 infection through angiotensin-converting enzyme 2 (ACE2) induces an uncontrolled cytokine storm, characterized by high levels of IL-1β, IL-6, IL-12, IL-18, TNF-α, and IFN-γ. The authors underscore that key chemokines, such as CCL2, CCL3, CCL5, CXCL8, and CXCL10, serve as critical prognostic factors to identify high-risk patients. Moving from acute risk to chronic sequelae, Quiroga et al. link persistent lung impairment in severe COVID-19 survivors to elevated CXCL9/CXCL10 levels, but not classical pro-inflammatory cytokines, like IL-12, IL-6, IL-1β, and TNF-α. CXCL9 and CXCL10 drive B cell differentiation and sustain chronic inflammation, positioning the CXCL9/CXCL10–CXCR3 axis as a viable therapeutic target. Therefore, both studies offer interesting therapeutic avenues based on the identified mechanisms of cytokines in viral diseases/pathogenesis.

Further expanding our understanding of antiviral immune regulation, Danzer et al. addressed the anti-inflammatory role of bitter taste receptors (TAS2Rs) on human peripheral blood mononuclear cells (PBMCs) stimulated with a peptide pool from SARS-CoV-2. This peptide pool increased the expression of several pro-inflammatory cytokines, like IL-2, IL-7, and IL-12A, and chemokines. Focusing on the induced chemokines, the authors demonstrated the inhibitory activity of Homoeriodictyol (a TAS2Rs ligand) in the PBMCs stimulated with the peptide pool from SARS-CoV-2. Through this, the authors identify the broadly tuned TAS2R14 as a promising target for anti-inflammatory regulation during viral infections. Addressing these shared respiratory cascades from a therapeutic perspective, Cao et al. systematically summarize the molecular mechanisms of IL-37, highlighting its dual intracellular and extracellular protective role in mitigating virus-induced hyperinflammation, alleviating asthma by inhibiting Th2/Th17 responses, and suppressing progression in non-small cell lung cancer and pulmonary fibrosis. These studies further emphasize that dysregulated cytokine networks not only shape acute disease severity but may also influence long-term clinical outcomes.

Exploring another dimension of cytokine-driven hyperinflammation, Zhang et al. utilize mass cytometry to map pediatric recovery in Histoplasma-associated hemophagocytic lymphohistiocytosis. By this means, the authors track a crucial metabolic shift from a glycolysis-driven inflammatory state to an oxidative, immunoregulatory M2 macrophage phenotype associated with reduced levels of proinflammatory cytokines, and resolution. Finally, Nuyttens et al. reveal that TNF-α-driven systemic inflammatory response syndrome (SIRS) causes fatal metabolic failure by impairing the hepatic malate-aspartate shuttle. This study provides new insights into the mechanisms underlying organ dysfunction and identifies metabolic restoration as a promising therapeutic strategy to prevent systemic collapse.

Collectively, the studies in this Research Topic illustrate that cytokines should no longer be viewed as isolated inflammatory mediators but as highly interconnected regulatory networks that bridge immunity, metabolism, tissue repair, vascular biology, and host-pathogen interactions. By synthesizing mechanistic insights with clinically actionable biomarkers, computational immune profiling, and innovative therapeutics, these contributions underscore the pivotal role of cytokine biology in advancing precision medicine. As our understanding of these networks evolves, the convergence of immunology, systems biology, and artificial intelligence will be essential to predict disease trajectories, personalize therapeutic interventions, and restore immune homeostasis across a diverse spectrum of human pathologies.

